# Effects of Temperature on Life-History Traits of *Paralipsa gularis* (Zeller) (Lepidoptera: Pyralidae), a Newly Emerged Maize Pest from the Border Areas Between China and Southeast Asian Countries

**DOI:** 10.3390/insects17050485

**Published:** 2026-05-09

**Authors:** Shuang Chen, Limei He, Xianming Yang, Guodong Kang, Lin Yang, Sengsathith Phalakhone, Xincheng Zhao, Kongming Wu

**Affiliations:** 1 Henan International Joint Laboratory of Green Pest Control, College of Plant Protection, Henan Agricultural University, Zhengzhou 450046, China; c860994099@163.com (S.C.); xincheng@henau.edu.cn (X.Z.); 2 State Key Laboratory for Biology of Plant Diseases and Insect Pests, Institute of Plant Protection, Chinese Academy of Agricultural Sciences, Beijing 100193, China; yangxianming@caas.cn (X.Y.); kanggd95@163.com (G.K.); yang_lin1996@126.com (L.Y.); 3 Institute of Urban Agriculture, Chengdu Agricultural Science and Technology Center, Chinese Academy of Agricultural Sciences, Chengdu 610299, China; helimei@caas.cn; 4 College of Plant Protection, Yunnan Agricultural University, Kunming 650201, China; 5 Plant Protection Center, Department of Agriculture, Ministry of Agriculture and Environment, Vientiane 01000, Laos; sthid63@gmail.com

**Keywords:** *Paralipsa gularis* (Zeller), temperature, biology, two-sex life table, demographic parameters

## Abstract

*Paralipsa gularis* (Zeller) (Lepidoptera: Pyralidae) is a storage pest native to Southeast Asia that has since spread to Northern Europe, North America, and other parts of Asia. In recent years, it has become an increasingly destructive pest in maize agroecosystems along the China–Southeast Asia border region, causing severe damage. Temperature is a key environmental factor influencing insect development, fecundity, and distribution, yet its effects on *P. gularis* remain unclear. In this study, we used an age-stage two-sex life table to evaluate the effects of temperature on the development duration, survival, and fecundity of *P. gularis*. We also determined the developmental threshold temperatures and effective accumulated temperatures for each life stage and developed a predictive model of developmental duration. Our results showed that temperature significantly affected the development duration across all stages, as well as larval and pupal survival rates and adult fecundity. Temperatures of 15–20 °C and 33–35 °C were unsuitable for population development, whereas 23–30 °C was optimal. At 15 and 35 °C, *P. gularis* failed to reach the pupal stage; at 33 °C, only a very few individuals reached adulthood. Between 18 and 30 °C, the development duration of each stage decreased with increasing temperature, with larval development ranging from 69.81 to 14.92 days. The optimal temperature was 28 °C, at which the insects exhibited high body weight, survival rate, intrinsic rate of natural increase (*r*), finite rate of increase (*λ*), and net reproduction rate (*R*_0_). The developmental threshold temperatures for eggs, larvae, pupae, and the overall generation were 17.33, 14.03, 11.72, and 13.11 °C, respectively, and the effective accumulated temperatures were 33.49, 242.51, 116.26, and 524.93 degree-days, respectively. This study provides a scientific basis and technical support for predicting population dynamics under varying climatic conditions and for developing effective control strategies for *P. gularis*.

## Simple Summary

*Paralipsa gularis* (Zeller), once a storage pest, has shifted to become a major pest in maize fields along the borders of China and Southeast Asian countries. In this study, we investigated the growth and development of *P. gularis* within a temperature range of 15–35 °C under laboratory conditions and determined its developmental threshold temperatures and effective accumulated temperatures. The results indicated that temperature significantly affected the developmental duration, survival rate, fecundity, and population growth parameters of *P. gularis*. The optimal range for these parameters was 23–30 °C, and 28 °C was most conducive to population growth. This study provides further information on the distribution area and population prediction of *P. gularis*, as well as on the development of a regional integrated pest management strategy for this pest.

## 1. Introduction

*Paralipsa gularis* (Zeller) (Lepidoptera: Pyralidae) is a storage pest native to Southeast Asia. It exhibits a wide geographic distribution, infesting not only Europe and North America but also Asian countries such as India, North Korea, and Japan [1,2,3]. It is widely distributed across provinces such as Jilin, Liaoning, Hebei, Henan, Shandong, Jiangsu, Zhejiang, Jiangxi, Sichuan, Fujian, Guizhou, and Yunnan in China [4]. In these regions, this pest was previously recorded primarily infesting stored maize, wheat, barley, soybeans, flax, and dried fruits with its larvae [3]. However, changes in climatic conditions and planting structures have significantly expanded the ecological niche of *P. gularis*, extending its range from storage environments to maize agroecosystems. Furthermore, *P. gularis* has gradually become a major pest in maize fields along the China–Southeast Asia border, posing a serious threat to maize production. In 2018, *P. gularis* was first reported as a new pest in maize production in Dehong Prefecture, Yunnan Province, China, where it damaged maize plants in the field by boring into maize ears during the late growth stage. In severe outbreaks, up to 20 individuals per maize ear were infested, with a plant infestation rate reaching 62.0%, triggering ear rot [5]. Subsequently, the pest infested the maize fields in the cities of Pu’er and Baoshan, Yunnan Province, China. The highest pest density and plant infestation rate reached 670.6 individuals per 100 plants and 94.4%, respectively. Furthermore, infestations occurred earlier in the growing stage and affected more parts of the maize plant, resulting in yield losses of up to 71.7% [6,7]. In addition, *P. gularis* infests maize ears in Laos, Vietnam, Myanmar, and the Guangxi and Guizhou Provinces in China [8].

Over the past 50 years, climate change has significantly affected the geographic distribution of insects. For example, as the climate has changed, major maize pests such as *Helicoverpa zea*, *Ostrinia nubilalis*, and *Diabrotica virgifera virgifera* have extended their overwintering regions further north in the United States [9], and the northern overwintering limit and potential range of *Riptortus pedestris* were also projected to expand [10]. Climate change also drives insects to invade and colonize new regions. For example, influenced by climate change, large lepidopteran species originally found in warm-temperate regions have invaded and established populations in northern Estonia [11], and climate change has led to a significant increase in the proportion of southern invasive species among diurnal lepidopterans in the coniferous forests of western Russia [12]. Although pest infestations are influenced by factors such as host and natural enemies, studies have shown that under identical host-feeding conditions, rising temperature is more likely to lead to pest infestations [13]. Furthermore, invasive species may exhibit a greater capacity to adapt to climate change than native species, as their high fecundity and abundance make them more damaging to crops [14,15]. As temperatures rise, temperature-driven changes are believed to alter ecosystem stability and influence the generation cycles of multivoltine insects [16]. Consequently, rising temperatures are likely to expand the global distribution of pests, trigger pest outbreaks, and ultimately increase crop damage.

Insects are ectothermic animals; their growth, development, fecundity, distribution, and life history strategies are all directly regulated by temperature [17]. The developmental rate of insects increases with rising temperature [18]. In the 1980s, studies revealed that the storage pest, *P. gularis*, exhibited remarkable adaptability to environmental changes. At 22–30 °C, its developmental duration gradually shortened; 30 °C was the optimal temperature for its growth and development, and its population showed a significant tendency to expand [3]. Currently, *P. gularis* has shifted from causing damage in storage facilities to causing damage in the maize fields. This behavioral change suggests that the current adaptability of *P. gularis* to environmental changes may differ from its adaptability as a storage pest. However, our understanding of *P. gularis* is currently limited to its biological characteristics, such as the host plants it infests and its distribution in the field [5,6,7]; its adaptive capacity in response to environmental changes remains unclear. Therefore, to prevent environmental changes from triggering pest outbreaks that could cause severe crop damage, it is necessary to assess the effects of temperature on *P. gularis*.

The age-stage, two-sex life table is an important method and technique for studying the effects of environmental factors on the survival, growth, development, and fecundity of insect populations and plays a crucial role in pest forecasting and control [19,20]. Here, we used the age-stage, two-sex life table to evaluate the effects of temperature on the developmental duration, larval survival rate, pupal survival rate, and adult fecundity. We also determined the developmental threshold temperatures and effective accumulated temperatures for each life stage of *P. gularis*. This study aims to elucidate the growth and development patterns of *P. gularis* at different temperatures, facilitating improved predictions of regional population dynamics and contributing to the development of integrated management strategies for *P. gularis*.

## 2. Materials and Methods

### 2.1. Insects

The insects were collected in Baozang Town, Jiangcheng County, Pu’er Prefecture, Yunnan Province, China (22°40′55.82″ N, 101°38′51.44″ E). Adults attracted overnight by a vertical searching light trap equipped with a 1000 W metal-halide lamp (Model JLZ1000BT, Shanghai Yaming Lighting Co., Ltd., Shanghai, China) were collected each morning and placed in a cylindrical plastic container (3000 mL) to allow them to lay eggs. A cotton ball soaked in 10% (*v*/*v*) honey water was placed at the bottom of the cylindrical plastic container to provide nutritional support for the adults, and a sterile gauze layer was placed on top to facilitate egg collection. The next day, the gauze containing the eggs was cut out and placed in a 50 mL centrifuge tube for hatching; a cotton ball moistened with clean water was placed at the opening of the tube to maintain humidity. The hatched larvae were placed individually in cylindrical plastic containers (25 mL) equipped with a square artificial diet (approximately 1.5 cm in length, width, and height) [21]. All larvae and adults were reared under environmental conditions of 26 ± 1 °C, 65% ± 10% relative humidity, and a 16:8 h light–dark cycle.

### 2.2. Experimental Design

The effects of different temperatures on the growth, development, survival, and fecundity of *P. gularis* were observed in artificial climate chambers (RXZ-500D, Ningbo Jiangnan Instrument Factory, Ningbo, China). Based on the field occurrence characteristics of *P. gularis*. and local climatic conditions [5,6,7,22], the experiment included 9 treatment groups set at 15, 18, 20, 23, 25, 28, 30, 33, and 35 °C, with a 16:8 h light-dark cycle, and 75% relative humidity.

Eggs laid by adults were placed in 50 mL centrifuge tubes and then placed in artificial climate chambers at different temperatures. Hatched larvae were individually transferred to 25 mL cylindrical plastic containers, which were covered with a layer of 120-mesh nylon netting to prevent escape, and numbered sequentially. The larvae were fed an artificial diet made primarily from soybean meal and wheat bran [21]. To prevent mold, spoilage, or dehydration of the artificial diet from influencing the trial results, it was replaced every 2–3 days. The growth, development, and mortality of *P. gularis* were observed and recorded daily. For each treatment, 80 eggs/larvae were observed, with the experiment repeated three times, for a total of 240 eggs or larvae. The larval instar was determined by observing the number of head capsules (molts) each day. The larval instar was equal to the number of head capsules plus one, i.e., newly hatched larvae were at the first instar, larvae entered the second instar after the first head capsule was observed, and the third instar after the second head capsule, etc. On the first day of the fifth instar, the larvae were weighed using an electronic balance (AR124CN, accuracy 0.0001 g, Ohaus Instruments (Changzhou) Co., Ltd., Changzhou, China). After pupation, the artificial diet was removed, the pupae were weighed on the third day, and any deformed pupae were observed and recorded.

After adult emergence, we observed and recorded adult deformities and sex, then paired them individually (♀:♂ = 1:1) and placed them in 450 mL cylindrical transparent plastic cups. We covered the cup openings with sterile, non-woven gauze to collect eggs. We also placed a cotton ball soaked in 10% (*v*/*v*) honey water at the bottom of the plastic cup to provide nutritional support for the adults and replaced it daily. The adults’ daily egg-laying and mortality were observed and recorded. The eggs laid by the adults into 6 cm × 8.5 cm transparent plastic resealable bags were collected and placed in the artificial climate chamber, and the hatching of the eggs was observed daily. Finally, the dead female moths were collected and dissected; mating status was determined by counting spermatozoa in the spermatophores, which enabled calculation of the females’ fecundity parameters and mating rates.

### 2.3. Methods for Calculating the Developmental Threshold Temperatures and Effective Accumulated Temperatures

Based on the effective accumulated temperature rule, the least-squares method was used to estimate the developmental threshold temperatures and effective accumulated temperatures for *P. gularis*. The formulas for calculating the developmental threshold temperatures and effective accumulated temperatures are as follows:

T=C+KV

$$C=\frac{\sum V^{2}\sum T-\sum V\sum VT}{n\sum V^{2}-{\left(\sum V\right)}^{2}}$$

$$K=\frac{n\sum VT-\sum V\sum T}{n\sum V^{2}-{\left(\sum V\right)}^{2}}$$

$$S_{c}=\sqrt{\frac{\sum {\left(T-T^{\prime }\right)}^{2}}{n-2}\left[\frac{1}{n}+\frac{V^{\prime 2}}{\sum {\left(V-V^{\prime }\right)}^{2}}\right]}$$

$$S_{k}=\sqrt{\frac{\sum {\left(T-T^{\prime }\right)}^{2}}{\left(n-2\right)\sum {\left(V-V^{\prime }\right)}^{2}}}$$

Note: *T*—environmental temperature; *T’*—mean of *T*; *C*—the developmental threshold temperatures; *K*—the effective accumulated temperatures; *V*—developmental rate (1/developmental duration); *V’*—mean of *V*; *n*—number of temperature treatment groups (in this study, *n* = 7); *S_c_*—standard deviation of the developmental threshold temperatures; *S_k_*—standard deviation of the effective accumulated temperatures.

### 2.4. Data Analysis

Data analysis and linear model fitting were conducted using Python version 3.14. The Kruskal–Wallis nonparametric test was used to analyze significant differences in the growth, development, and fecundity parameters of *P. gularis* at different temperatures; where significant differences were found, multiple comparisons were performed. The Mann–Whitney U nonparametric test was used to analyze sex differences in developmental duration and the weights of larvae and pupae at the same temperature. A general linear model was used to analyze the interaction between temperature and sex on the developmental durations of larvae, pupae, and adults, the total generation, and the fifth instar larval and pupal weights. One-way ANOVA was used to examine whether there were significant differences in larval survival rate, pupal survival rate, pupal deformity rate, and adult deformity rate across different temperatures; when significant differences were found, Duncan’s multiple range test was conducted. Proportional data were arcsine-square-root-transformed before a one-way ANOVA. The log-rank test was used in GraphPad Prism 10.1 (GraphPad Software Inc., San Diego, CA, USA) to analyze differences in survival curves and their linear trends for *P. gularis* at different temperatures. A linear model was used to fit the relationship between the developmental rate of *P. gularis* and temperature, and Pearson’s correlation coefficients were used to assess the significance of the relationship between observed and predicted developmental durations. We used TWOSEX-MSChart (Version 07/06/2024) to calculate the life tables (*l_x_*, *f_x_*, *m_x_*, and *l_x_m_x_*) and demographic parameters (*R*_0_, *T*, *r*, and *λ*) of *P. gularis* at different temperatures [20,23,24]. We used a paired bootstrap test with 100,000 replications in TWOSEX-MSChart for the precise estimation of the mean and standard error among the demographic parameters of *P. gularis* at different temperatures [24,25,26].

## 3. Results

### 3.1. The Effect of Temperature on Developmental Duration

**Figure 1:**
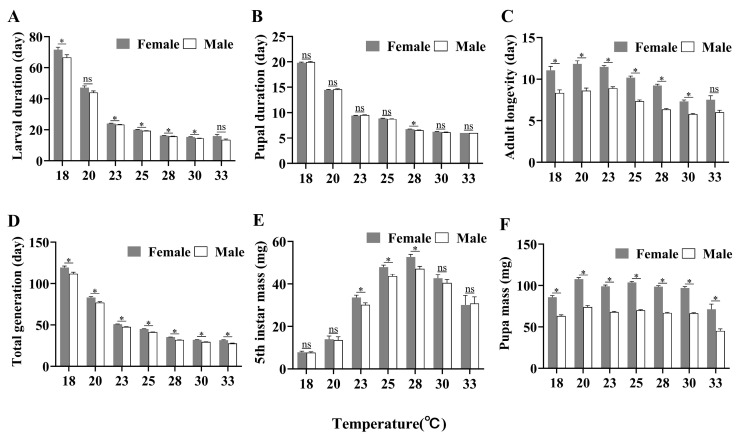
Mean duration (± SE) of developmental stages ((**A**) larval duration; (**B**) pupal duration; (**C**) adult longevity; (**D**) total longevity), fifth instar mass (**E**), and pupal mass (**F**) of female and male *Paralipsa gularis* (Zeller) at different temperatures. *: *p* < 0.05; ns: *p* > 0.05. Number of females/males in the figure: 54/41 (18 °C); 59/36 (20 °C); 115/101 (23 °C); 106/101 (25 °C); 112/111 (28 °C); 77/90 (30 °C); 2/10 (33 °C).

At 15 °C, the eggs of *P. gularis* failed to hatch normally; at 35 °C, the hatching rate was extremely low, with only 90 larvae emerging, none of which survived to the second instar. At 18–33 °C, *P. gularis* was able to develop into adults. Temperature had a significant effect on the developmental duration of each stage of *P. gularis* (egg: *H* = 1756.889, *df* = 7, *p* < 0.001; first instar larvae: *H* = 1239.391, *df* = 7, *p* < 0.001; second instar larvae: *H* = 1160.339, *df* = 6, *p* < 0.001; third instar larvae: *H* = 1183.536, *df* = 6, *p* < 0.001; fourth instar larvae: *H* = 1135.779, *df* = 6, *p* < 0.001; fifth instar larvae: *H* = 977.723, *df* = 6, *p* < 0.001; sixth instar larvae: *H* = 504.007, *df* = 6, *p* < 0.001; fifth–tenth instar larvae: *H* = 843.983, *df* = 6, *p* < 0.001; larval stage: *H* = 1021.637, *df* = 6, *p* < 0.001; pupal stage: *H* = 931.963, *df* = 6, *p* < 0.001; adult stage: *H* = 319.019, *df* = 6, *p* < 0.001; egg-pupa stage: *H* = 941.676, *df* = 6, *p* < 0.001; total generations: *H* = 934.371, *df* = 6, *p* < 0.001). Overall, the developmental durations of *P. gularis* at each stage shorten as temperature increases; at 30 and 33 °C, the developmental durations of the larvae, pupae, adults, and the total generation were significantly shorter than those at 18–28 °C. Larval developmental duration was shortest at 30 °C and longest at 18 °C, with durations of 14.92 days and 69.81 days, respectively. The developmental durations of eggs, pupae, adults, and the total generation were shortest at 33 °C: 2.00, 6.00, 6.25, and 28.17 days, respectively. The developmental durations for eggs, pupae, and total generation were longest at 18 °C, at 17.00, 19.86, and 116.21 days, respectively; the developmental duration for adults was longest at 20 °C, but there was no significant difference compared with those at 18 °C, at 10.60 days and 9.88 days, respectively (Table S1). The development durations of female larvae (*χ*^2^ = 11.620, *df* = 1, *p* < 0.001), adults (*χ*^2^ = 116.677, *df* = 1, *p* < 0.001), and total generation (*χ*^2^ = 51.418, *df* = 1, *p* < 0.001) were significantly longer than those of males. In contrast, there was no significant difference in the pupal stage between males and females (*χ*^2^ = 0.013, *df* = 1, *p* = 0.910) (Figure 1A–D). In addition, temperature affected the progression of instars in *P. gularis*. At a low temperature of 18 °C, the number of instars was highest, with even a single tenth instar larva observed. As the temperature gradually increased, the number of instars decreased; at 20 °C, ninth instar larvae were observed, and at 23 °C, seventh instar larvae were observed. When the temperature rose to 28 °C, the majority of larvae developed to the fifth instar and began pupation, with only 20 larvae reaching the sixth instar. However, as the temperature continued to rise, the number of instars again increased; at 33 °C, 3 ninth instar larvae were observed (Table S1).

The weight of fifth instar larvae and pupae at different temperatures differed significantly (fifth instar mass: *H* = 914.976, *df* = 6, *p* < 0.001; pupa mass: *H* = 170.572, *df* = 6, *p* < 0.001). The weight of fifth instar larvae was highest at 28 °C (49.72 mg) and lowest at 18 °C (6.86 mg), while the weight of pupae was highest at 20 °C (94.08 mg) and lowest at 33 °C (48.09 mg) (Table S1). The weight of fifth instar larvae did not differ significantly between sexes (*χ*^2^ = 2.355, *df* = 1, *p* = 0.125), but the weight of female pupae was significantly higher than that of male pupae (*χ*^2^ = 402.212, *df* = 1, *p* < 0.001) (Figure 1E,F). Temperature and sex had significant interactive effects on development durations of larvae (*χ*^2^ = 24.196, *df* = 6, *p* < 0.001), pupae (*χ*^2^ = 13.655, *df* = 6, *p* = 0.034), adults (*χ*^2^ = 20.014, *df* = 6, *p* = 0.003), and total generation (*χ*^2^ = 23.237, *df* = 6, *p* < 0.001) and pupal weight (*χ*^2^ = 15.787, *df* = 6, *p* = 0.015), but no significant interactive effect was observed for fifth instar larval weight (*χ*^2^ = 6.169, *df* = 6, *p* = 0.405).

### 3.2. Life Table Parameters on Different Temperatures

**Figure 2:**
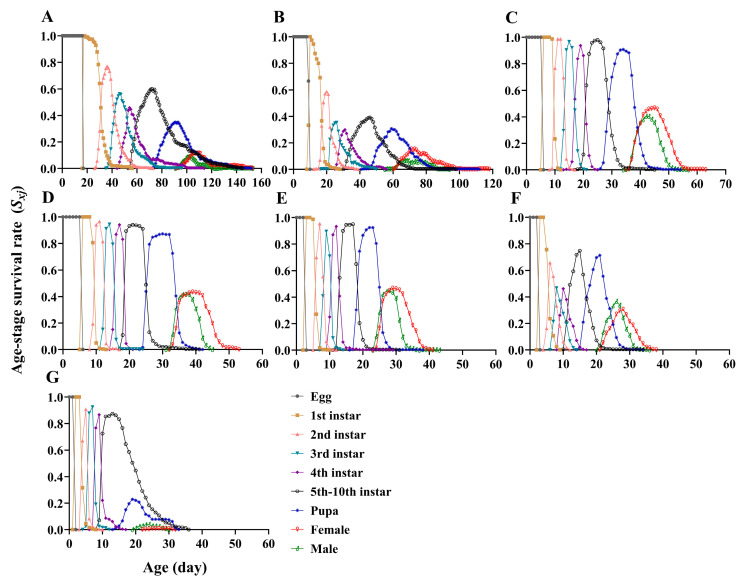
Age-stage survival rate (*S_xj_*) of *Paralipsa gularis* (Zeller) at different temperatures. (**A**) 18 °C; (**B**) 20 °C; (**C**) 23 °C; (**D**) 25 °C; (**E**) 28 °C; (**F**) 30 °C; (**G**) 33 °C.

**Figure 3:**
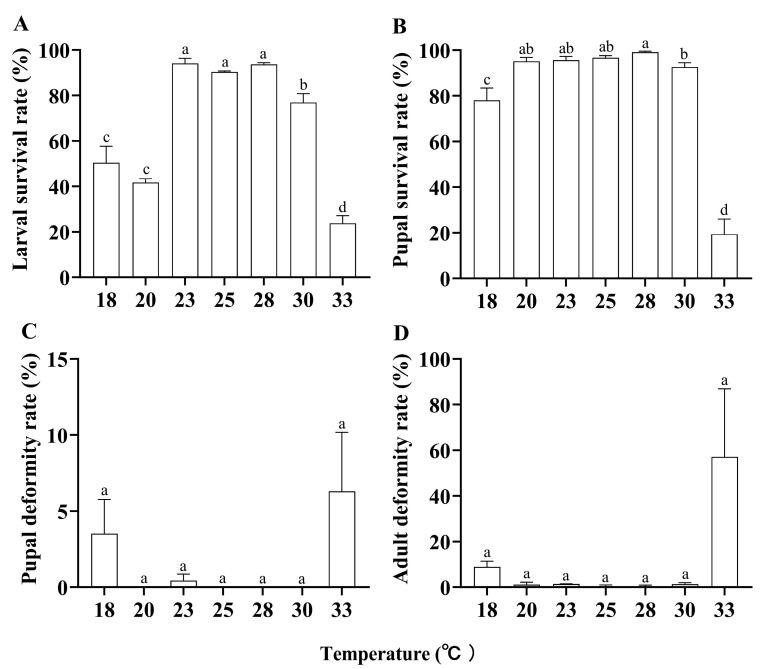
Life parameters of *Paralipsa gularis* (Zeller) at different temperatures. (**A**) Larval survival rate; (**B**) pupal survival rate; (**C**) pupal deformity rate; (**D**) adult deformity rate. Data in the figure are given as the mean ± SE. Different lowercase letters indicate significant differences between temperatures (*p* < 0.05).

The age-stage survival rate (*S_xj_*) of *P. gularis* showed marked differences at different temperatures (Figure 2). The larval survival rates differed significantly at different temperatures (*F*_6,14_ = 64.862, *p* < 0.001). At 23–28 °C, there were no significant differences in larval survival rates, but they were significantly higher than at other temperatures. Specifically, the larval survival rate was highest at 23 °C (94.17% ± 2.20%) and lowest at 33 °C (23.75% ± 3.31%). There was also a significant difference in pupal survival rates (*F*_6,14_ = 46.735, *p* < 0.001). Pupal survival rate was highest at 28 °C (99.11% ± 0.44%) and lowest at 33 °C (19.35% ± 6.65%). Pupal deformity rate (*F*_6,14_ = 2.541, *p* = 0.071) and adult deformity rate (*F*_6,14_ = 2.795, *p* = 0.053) showed no significant differences at different temperatures. However, at 33 °C, the deformity rates for pupae (6.30% ± 3.87%) and adults (57.14% ± 29.74%) were higher than at other temperatures, with 18 °C showing the next highest rates (Figure 3). Consequently, lower and higher temperatures affected the survival rates of larvae and pupae, as well as the deformity rates of pupae and adults.

**Figure 4:**
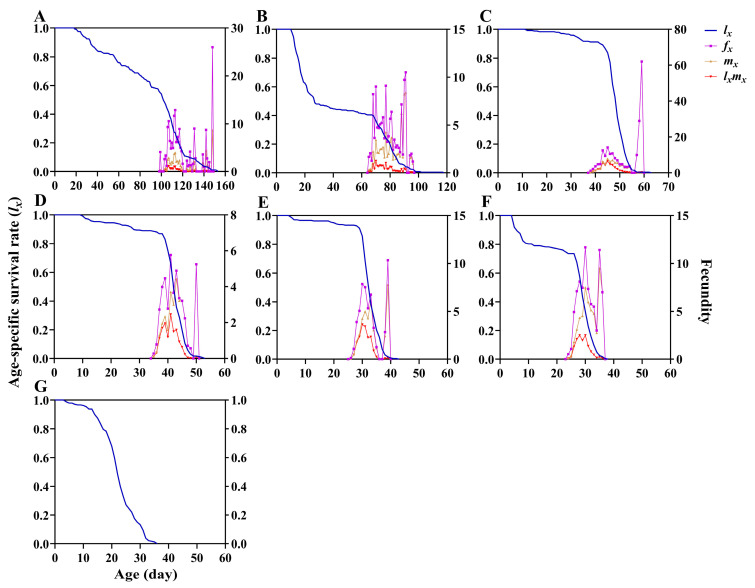
Age-specific survival rate (*l_x_*), age-specific fecundity of female adults (*f_x_*), age-specific fecundity (*m_x_*), and age-specific maternity (*l_x_m_x_*) of *Paralipsa gularis* (Zeller) individuals at different temperatures. (**A**) 18 °C; (**B**) 20 °C; (**C**) 23 °C; (**D**) 25 °C; (**E**) 28 °C; (**F**) 30 °C; (**G**) 33 °C.

**Figure 5:**
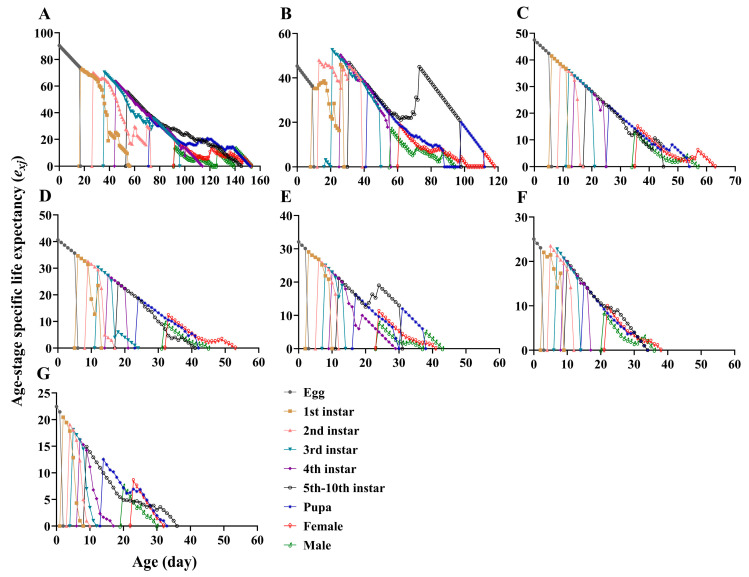
Age-stage specific life expectancy (*e_xj_*) of *Paralipsa gularis* (Zeller) individuals at different temperatures. (**A**) 18 °C; (**B**) 20 °C; (**C**) 23 °C; (**D**) 25 °C; (**E**) 28 °C; (**F**) 30 °C; (**G**) 33 °C.

The survival analysis revealed highly significant differences in age-specific survival rates (*l_x_*) at different temperatures (*χ*^2^ = 1597, *df* = 6, *p* < 0.0001), as well as a significant linear trend (*χ*^2^ = 1416, *df* = 1, *p* < 0.0001) (Figure 4). At 18, 20, 23, 25, 28, and 30 °C, the maximum value of age-specific fecundity of female adults (*f_x_*) was 26.00, 10.50, 62.00, 5.76, 10.33, and 11.68, respectively. The maximum values of age-specific fecundity (*m_x_*) occurred on days 148, 91, 45, 43, 39, and 35, respectively, while the maximum values of age-specific maternity (*l_x_m_x_*) occurred on days 107, 70, 45, 41, 30, and 30, respectively. The age-stage-specific life expectancy (*e_xj_*) curve showed that the maximum life expectancy of *P. gularis* occurred during the egg stage at all temperatures, with the highest value at 18 °C and the lowest at 33 °C. The life expectancy of *P. gularis* decreases with increasing age, while it shows an increase during the fifth–tenth instar larval stage, the pupal stage, and the adult stage. The life expectancy of *P. gularis* is the longest at 18 and 20 °C, while it is the shortest at 33 °C (Figure 5).

Temperature significantly affected the fecundity and life table parameters of *P. gularis* (Table 1). At 33 °C, the female adults died before laying eggs, and very few individuals (two individuals) reached the female adult stage. At 18–30 °C, the pre-oviposition period (*χ*^2^ = 60.065, *df* = 5, *p* < 0.001), oviposition period (*χ*^2^ = 24.131, *df* = 5, *p* < 0.001), and fecundity (*χ*^2^ = 30.087, *df* = 5, *p* < 0.001) of female *P. gularis* all showed significant differences. As the temperature increased, the pre-oviposition period gradually shortened. The pre-oviposition period was shortest at 30 °C, lasting 3.90 days. The oviposition period and fecundity at 23 °C were significantly higher than at other temperatures. At 18 °C, the net reproduction rate (*R*_0_), intrinsic rate of natural increase (*r*), and finite rate of increase (*λ*) were lowest, while the mean generation time (*T*) was longest (112.77 days). The mean generation time (*T*) decreased gradually with increasing temperature, reaching a minimum of 29.83 days at 30 °C. At 28 °C, the intrinsic rate of natural increase (*r*) and finite rate of increase (*λ*) were highest, and the net reproduction rate (*R*_0_) was second only to that at 23 °C, indicating the fastest population growth; this temperature condition was most favorable for population growth. The results of the life table parameters indicate that the growth capacity of the *P. gularis* population, from strongest to weakest, was 28 °C > 30 °C > 23 °C > 25 °C > 20 °C > 18 °C.

### 3.3. Developmental Threshold Temperatures and Effective Accumulated Temperatures

The developmental threshold temperatures and effective accumulated temperatures for each developmental stage of *P. gularis* vary, and these differ between sexes as well. Specifically, the developmental threshold temperatures and effective accumulated temperatures for eggs were 17.33 °C and 33.49 degree-days, respectively; for larvae, they were 14.03 °C and 242.51 degree-days; for pupae, they were 11.72 °C and 116.26 degree-days; and for total generation, they were 13.11 °C and 524.93 degree-days. The developmental threshold temperatures of female eggs, larvae, pupae, and total generation were 17.33, 14.09, 11.58, and 12.73 °C, respectively, with effective accumulated temperatures of 33.49, 248.11, 117.97, and 571.60 degree-days, respectively. For male moths, the developmental threshold temperatures for eggs, larvae, pupae, and total generation were 17.34, 14.02, 11.88, and 13.05 °C, respectively, with effective accumulated temperatures of 33.48, 232.95, 114.43, and 507.08 degree-days, respectively (Table 2).

### 3.4. Mathematical Modeling and Statistical Testing of Developmental Rate as Temperatures Change

**Figure 6:**
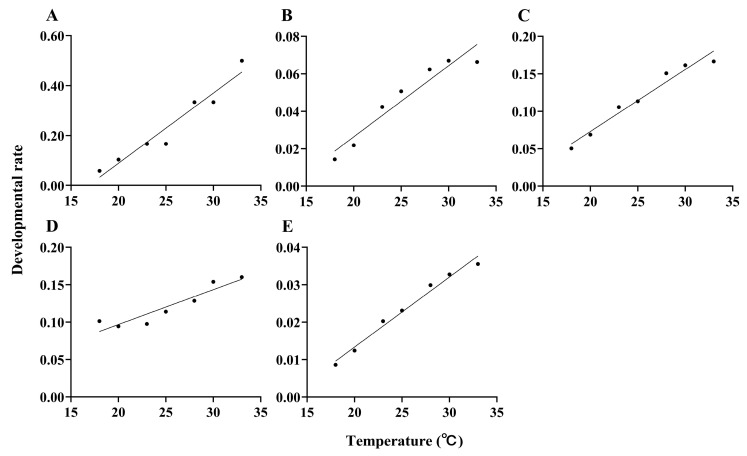
The relationship between developmental rates and temperature at various life stages of *Paralipsa gularis* (Zeller). (**A**) egg; (**B**) larva; (**C**) pupa; (**D**) adult; (**E**) generation.

A linear model was applied to fit the relationship between developmental rate and temperature, yielding the following model equation for eggs: *V* = 0.028*T* − 0.473 (*R*^2^ = 0.941, *F*_1,5_ = 79.689, *p* < 0.001); for larvae: *V* = 0.004*T* − 0.050 (*R*^2^ = 0.921, *F*_1,5_ = 58.522, *p* < 0.001); pupae: *V* = 0.008*T* − 0.093 (*R*^2^ = 0.962, *F*_1,5_ = 127.466, *p* < 0.001); adults: *V* = 0.005*T* + 0.004 (*R*^2^ = 0.871, *F*_1,5_ = 33.910, *p* = 0.002); for the total generation: *V* = 0.002*T* − 0.024 (*R*^2^ = 0.982, *F*_1,5_ = 271.985, *p* < 0.001) (Figure 6). The coefficient of determination (*R*^2^) for the models of each developmental stage was over 0.8, and the observed values were significantly correlated with the model predictions (*p* < 0.05).

## 4. Discussion

All insects require specific temperature conditions to survive, ensuring that individuals can grow and develop normally and that populations can be sustained. Within the optimal temperature range, the developmental duration of most insects is positively correlated with temperature; a moderate increase in temperature promotes population expansion [27,28,29]. Faster development rates may benefit insects by reducing the time they spend in vulnerable stages, when they are susceptible to attacks by predators, predatory wasps, and entomopathogens [30]. In this study, the developmental rates of *P. gularis* at all stages exhibited a linear relationship with temperature; at 18–33 °C, developmental rates gradually increased and generation cycles progressively shortened. This is consistent with previous findings showing that the developmental duration of *Spodoptera frugiperda* (J.E. Smith) shortened with rising temperature at 18–32 °C, and that the developmental duration of *Ostrinia furnacalis* (Guenée) shortened with rising temperature at 20–32 °C [19,31]. However, the temperature that exceeds or falls below optimal ranges can reduce insect fitness or even lead to death; this phenomenon was observed in lepidopteran insects such as *S. frugiperda* and *Epiphyas postvittana* [32,33]. Our study also found that the eggs of *P. gularis* failed to hatch at a low temperature of 15 °C, and the larvae were unable to develop to the second instar at a high temperature of 35 °C; survival rates at 18 and 33 °C were significantly lower than those at 23–30 °C. Furthermore, when the temperature is unsuitable for insect growth and development, the number of larval instars increases [34,35]. The number of instars in *P. gularis* increased at low and high temperatures, indicating that these temperatures were unfavorable for its growth and development. In addition, the developmental duration of female *P. gularis* was longer than that of males, and male pupae emerged earlier. This phenomenon was similar to that observed in pyralidae such as *O. furnacalis* and *Loxostege sticticalis* [19,36], but contrasts with the pattern seen in noctuidae like *S. frugiperda*, *Helicoverpa armigera*, and *Spodoptera litura*, where male development duration was significantly longer than that of females, and female pupae emerged earlier [37,38,39]. This might be because insects require a certain amount of heat accumulation to complete their life history, and females demand more heat than males [40]. This was consistent with our findings that the effective accumulated temperatures for female larvae, pupae, adults, and generations of *P. gularis* were higher than those for males.

Previous studies revealed that among 67 insect species, 18% exhibited a significant increase in body size with rising temperature, while 7% reached their maximum body size at moderate temperature [41,42]. In this study, the weight of the fifth instar larvae of *P. gularis* increased with rising temperature, peaking at 28 °C before declining, while pupae exhibited higher weights at 20–30 °C compared with 18 and 33 °C. This was consistent with the phenomenon observed in *O. furnacalis*, which exhibited higher body weights at higher temperatures [41]. In general, the heavier the larvae and pupae of insects are, the greater their fecundity and adaptability, and the higher their egg-laying capacity [41]. For example, the egg-laying rate of *Plutella xylostella* (Linnaeus) was positively correlated with pupal weight [43]; heavier and medium-sized female *Copitarsia decolora* (Guenée) laid more eggs [44]; and smaller *Frankliniella occidentalis* exhibited lower fecundity [45]. Our research results also confirmed this phenomenon: at 28 °C, the fifth instar larvae and pupae of *P. gularis* had higher weights, and at this temperature, *P. gularis* exhibited the highest intrinsic rate of natural increase (*r*), and finite rate of increase (*λ*), with the net reproduction rate (*R*_0_) second only to that at 23 °C. In summary, temperatures ranging from 23 to 30 °C were suitable for the growth and development of *P. gularis*, with 28 °C being the most optimal for the growth of the *P. gularis* population. However, all females in this study were unmated, and the eggs they laid were unfertilized and non-viable. The life table parameters calculated from these non-viable eggs—including adult fecundity, intrinsic rate of natural increase (*r*), finite rate of increase (*λ*), and the net reproduction rate (*R*_0_)—may differ from those obtained under mated conditions. Therefore, the current life table parameters are provided for reference only.

Mating is a critical stage in insect population growth and is closely related to subsequent behaviors such as oviposition and hatching. Temperature can significantly influence the mating behavior of most insects. Studies have shown that the mating rate of *Maruca vitrata* (Fabricius) peaks at 22 °C [46]; 25 °C is conducive to the mating of *Plutella xylostella* [47]; and the mating activity of *Chilo suppressalis* (Walker) is most pronounced between 28 °C and 31 °C, with the mating rate dropping significantly above 31 °C [48]. In this study, no *P. gularis* mated under any constant-temperature conditions. However, the *P. gularis* we captured from the wild were mating, which may be related to temperature fluctuations in the natural environment. The growth, development, and mating behaviors of insects under fluctuating temperatures may differ from those under constant-temperature conditions [49,50,51]. Therefore, it is necessary to investigate the effects of fluctuating temperatures on *P. gularis*. Beyond temperature, the success rate of insect mating is also influenced by factors such as humidity, photoperiod, host plants and their volatiles, rearing conditions, sex ratio, and pheromones [52,53]. For example, humidity is positively correlated with the mating frequency of *O. furnacalis*; high-humidity environments enhance its mating activity [54]. *S*. *litura* feeds on tobacco mate the most frequently and has a higher probability of multiple matings, while those feeding on Chinese cabbage exhibit the longest mating duration [55]. Volatiles from cowpea flowers significantly promote courtship and mating behavior in both male and female *M. vitrata* [46]. *Chelonus formosanus* Sonan exhibits the highest mating rate when the sex ratio is 1:7; as the rearing space increases from 348 cm^3^ to 1920 cm^3^, the mating rate gradually decreases [56]. We previously conducted experiments on the outdoor rearing of *P. gularis* using insect rearing cages of varying sizes. Preliminary findings indicate that on rainy days, regardless of cage size, a small number of individuals mate and lay eggs. Based on this observation, we tentatively hypothesize that humidity may be related to the mating behavior of *P. gularis*. However, the natural outdoor environment is relatively complex. Therefore, in subsequent artificial rearing, we need to simulate the natural environment as closely as possible, taking into account factors such as humidity, air circulation, and temperature fluctuations, as well as the interactions among these factors. This will address the challenges of artificial rearing of *P. gularis* and provide a theoretical foundation for population forecasting and integrated pest management.

The shift in *P. gularis* from causing damage in storage facilities to causing damage in maize fields represents a significant change in its ecological niche; this phenomenon is likely closely related to host plants and the environment [5]. Host plants are among the factors influencing changes in their habitat; they serve not only as a food source for the pest but also as a component of its sheltering environment. In storage facilities, host plants are limited and monotonous, whereas in maize agroecosystems, they are complex and diverse, potentially providing abundant food for *P. gularis*. Furthermore, under the same host conditions, rising temperature is more likely to enhance the insect’s fitness [13]. This study found that *P. gularis* exhibited strong temperature adaptation, which may serve as a key foundation for its ecological expansion and host shift [3]. Evidence indicates that compared with enclosed warehouse environments, open warehouse conditions favor the growth and development of *P. gularis* [3]. Similarly, temperature in natural field environments fluctuates more than in storage facilities, which may be more conducive to the development of the *P. gularis* population. Global warming has led to a general northward expansion of insect distribution ranges [57]. *P. gularis* adults exhibited strong adaptability to temperature and had migrated from the Indo-China Peninsula into Yunnan Province, China; trajectory analysis indicated that they primarily migrate northward [58]. As China’s primary grain-producing region, the north has abundant host plants. Therefore, the border areas between China and Southeast Asian countries are not only a suitable habitat for *P. gularis* but may also serve as a key source of pests for its northward expansion, posing a potential threat to safe production in China’s major grain-producing areas. Considering this, it is essential to further strengthen research on the occurrence dynamics and control technologies of *P. gularis* to effectively prevent and control the damage it causes.

Many pests and diseases exhibit characteristics of large-scale migration and epidemics; controlling them requires establishing a system based on regional monitoring, early warning, and control [59]. Currently, *P. gularis* primarily occurs in the warm, humid border regions of southwestern China, such as the provinces of Guangxi and Yunnan. These areas border Southeast Asian countries, including Myanmar, Laos, and Vietnam. Influenced by monsoons, populations of *P. gularis* migrate from Southeast Asian countries into southwestern China’s border areas during the spring. Consequently, the border areas face significant selective pressure from *P. gularis* infestations, making them a priority for integrated pest management efforts. Population monitoring and early warning form the foundation of pest management. For *P. gularis* adults in the China–Southeast Asia border areas, we can integrate large-scale radar monitoring with small-scale monitoring using high-altitude lights, ground lights, and sex pheromone traps. This integrated approach enables precise localization and quantification of adult migration patterns, thereby controlling population growth in year-round outbreak areas along the China–Southeast Asia border and managing populations migrating from abroad. This enables source control and minimizes the number of moths migrating to the Yangtze River region and northern areas. For larvae occurring in fields, we can predict the emergence periods, population sizes, and distribution areas of *P. gularis* at each developmental stage based on the developmental threshold temperatures and effective accumulated temperatures. By promptly issuing early warning information, we enable grassroots plant protection personnel to conduct precise field surveys, shifting from reactive response to proactive monitoring. Subsequently, based on actual field conditions, we can promptly employ chemical control, biological control, bait-based control using host plants, and planting Bt crops to reduce the initial population density at the source. Central and northern China should prioritize prevention, establish pest-monitoring and early-warning systems in advance, and intensify monitoring efforts to transition from reactive control upon detection to proactive control based on early warnings.

## 5. Conclusions

The border areas between China, Myanmar, Laos, and Vietnam serve as a migration pathway and year-round breeding ground for various important agricultural pests. *Paralipsa gularis* (Zeller), previously documented as a storage pest, has gradually emerged as a major pest during the late growth stages of maize in these areas. Here, we used the age-stage, two-sex life table to investigate how the growth, development, and fecundity of *P. gularis* respond to temperature fluctuations. The results indicated that *P. gularis* exhibited strong temperature adaptation. With global warming, our findings contribute to understanding the interactions between pests and the environment, providing theoretical support for the development of pest population forecasting and early warning technologies, as well as the establishment of a cross-border integrated pest management system.

## Figures and Tables

**Table 1:** The fecundity and life table parameters of *Paralipsa gularis* (Zeller) individuals at different temperatures.

Parameters	18 °C	20 °C	23 °C	25 °C	28 °C	30 °C
Pre-oviposition period (day)	6.93 ± 0.34 c	5.28 ± 0.31 b	5.05 ± 0.22 b	4.90 ± 0.23 b	4.09 ± 0.21 a	3.90 ± 0.19 a
Oviposition period (day)	2.28 ± 0.23 a	3.00 ± 0.33 a	3.55 ± 0.16 b	2.89 ± 0.19 a	2.93 ± 0.19 a	2.66 ± 0.22 a
Fecundity (*N*)	61.72 ± 6.62 a	70.67 ± 8.31 a	93.08 ± 5.92 b	52.27 ± 4.70 a	56.35 ± 5.05 a	55.91 ± 5.57 a
Net reproductive rate *R*_0_ (offspring/individual)	11.06 ± 1.92 c	12.66 ± 2.28 bc	41.11 ± 3.93 a	13.89 ± 1.94 bc	17.61 ± 2.30 b	13.86 ± 2.09 bc
Mean generation time *T* (d)	112.77 ± 1.08 a	76.58 ± 1.21 b	45.94 ± 0.31 c	41.46 ± 0.36 d	31.62 ± 0.22 e	29.83 ± 0.30 f
Intrinsic rate of natural increase *r* (d^−1^)	0.0213 ± 0.0016 e	0.0332 ± 0.0024 d	0.0809 ± 0.0023 b	0.0635 ± 0.0035 c	0.0907 ± 0.0044 a	0.0881 ± 0.0053 ab
Finite rate of increase *λ* (d^−1^)	1.0215 ± 0.0017 d	1.0337 ± 0.0024 c	1.0843 ± 0.0025 a	1.0655 ± 0.0037 b	1.0950 ± 0.0048 a	1.0921 ± 0.0057 a

Note: Data in the table are given as the mean ± SE. Different lowercase letters on the same row indicate significant differences between temperatures (*p* < 0.05).

**Table 2:** Developmental threshold temperatures and effective accumulated temperatures of different developmental stages of *Paralipsa gularis* (Zeller).

Parameters	Developmental Threshold Temperature/°C			Effective Accumulated Temperature/Degree-Days		
	Female	Male	Total	Female	Male	Total
Egg	17.33 ± 1.04	17.34 ± 1.04	17.33 ± 1.04	33.49 ± 3.75	33.48 ± 3.75	33.49 ± 3.75
1st instar	13.33 ± 1.90	13.80 ± 1.19	14.44 ± 1.09	50.81 ± 7.51	46.60 ± 4.45	43.68 ± 3.99
2nd instar	19.04 ± 1.71	15.46 ± 0.73	15.07 ± 0.68	16.16 ± 3.59	29.52 ± 1.95	31.51 ± 1.88
3rd instar	14.90 ± 1.14	14.71 ± 0.82	14.98 ± 1.42	31.74 ± 3.14	31.81 ± 2.23	31.91 ± 3.97
4th instar	14.41 ± 1.14	13.87 ± 0.88	14.39 ± 0.89	34.01 ± 3.25	35.42 ± 2.50	34.17 ± 2.53
5th instar	18.60 ± 3.67	19.08 ± 4.64	18.56 ± 4.05	26.18 ± 12.83	26.40 ± 18.07	27.40 ± 14.91
5th–10th instar	15.90 ± 3.43	14.46 ± 2.15	15.53 ± 3.15	80.49 ± 27.07	79.73 ± 14.58	78.86 ± 23.45
Larval stage	14.09 ± 1.85	14.02 ± 1.07	14.03 ± 1.60	248.11 ± 37.84	232.95 ± 20.25	242.51 ± 31.70
Pupa	11.58 ± 1.23	11.88 ± 1.33	11.72 ± 1.28	117.97 ± 12.85	114.43 ± 13.77	116.26 ± 13.29
Adult	1.47 ± 5.15	−2.05 ± 5.71	2.58 ± 3.98	225.55 ± 47.92	195.06 ± 40.21	187.21 ± 32.15
Egg-pupa	13.82 ± 1.31	13.97 ± 0.80	14.00 ± 0.86	405.04 ± 42.72	381.56 ± 24.77	386.59 ± 26.96
Total generation	12.73 ± 1.26	13.05 ± 0.93	13.11 ± 0.80	571.60 ± 53.53	507.08 ± 35.68	524.93 ± 31.83

## Data Availability

The original contributions presented in this study are included in the article/Supplementary Material. Further inquiries can be directed to the corresponding author.

## Supplementary Materials

The following supporting information can be downloaded at: https://www.mdpi.com/article/10.3390/insects17050485/s1, Table S1: Developmental duration of developmental stages of *Paralipsa gularis* (Zeller) at different temperatures.
