# Supplementary material for: Effects of Temperature on Life-History Traits of Paralipsa gularis (Zeller) (Lepidoptera: Pyralidae), a Newly Emerged Maize Pest from the Border Areas Between China and Southeast Asian Countries
Source: Insects. 2026 May 9;17(5):485. doi: 10.3390/insects17050485 (PMC13207621; doi:10.3390/insects17050485)
Supplement: Supplementary file 1 [file insects-17-00485-s001.zip › insects-4259579-supplementary.pdf]

Supplementary Materials:

Table S1. Developmental duration of developmental stages of *Paralipsa gularis* (Zeller) at different temperatures.

Parameters	18 °C	20 °C	23 °C	25 °C	28 °C	30 °C	33 °C	35 °C
Egg (day)	17.00±0.00(240)f	9.67±0.03(240)e	6.00±0.00(240)d	6.00±0.00(237)d	3.00±0.00(240)b	3.00±0.00(234)b	2.00±0.00(240)a	4.00±0.00(90)c
1st instar (day)	14.95±0.22(213)g	7.97±0.13(149)f	4.23±0.03(240)e	3.84±0.03(230)d	3.30±0.04(233)c	3.14±0.06(198)b	2.34±0.04(232)a	3.00±0.00(2)b
2nd instar (day)	11.81±0.30(186)f	6.66±0.26(119)e	3.56±0.04(237)d	3.08±0.03(225)c	2.36±0.03(232)b	2.32±0.06(187)b	1.74±0.03(231)a	
3rd instar (day)	11.20±0.36(174)f	6.74±0.26(112)e	3.78±0.05(236)d	3.04±0.02(223)c	2.19±0.03(231)b	2.00±0.04(186)a	2.12±0.03(228)b	
4th instar (day)	9.62±0.35(163)f	6.53±0.15(107)e	3.78±0.03(235)d	3.03±0.02(223)c	2.63±0.03(229)b	2.02±0.04(184)a	1.98±0.03(212)a	
5th instar (day)	9.14±0.23(154)f	6.51±0.17(105)e	6.63±0.11(228)d	2.22±0.28(219)a	5.03±0.06(226)c	3.24±0.11(184)b	2.89±0.07(189)b	
6th instar (day)	11.18±0.21(142)f	8.61±0.25(94)e	6.42±0.13(55)d	4.57±0.07(216)b	5.65±0.35(20)c	4.13±0.13(95)a	4.15±0.13(112)a	
7th instar (day)	11.85±0.41(52)	8.82±0.27(34)	9.00±1.50(3)	7.00(1)	-	-	4.30±0.28(30)	
8th instar (day)	13.19±0.94(16)	7.50±1.55(4)	-	-	-	-	5.11±0.59(9)	
9th instar (day)	13.33±1.67(3)	12.00(1)	-	-	-	-	5.00±0.00(3)	
10th instar (day)	17.00(1)	-	-	-	-	-	-	
5th–10th instar (day)	26.21±0.75(121)f	17.87±0.44(100)e	8.29±0.13(226)d	6.80±0.07(214)b	5.54±0.07(225)a	5.44±0.09(180)a	7.16±0.19(57)c	
Larval stage (day)	69.81±1.13(121)f	45.88±0.81(100)e	23.64±0.14(226)d	19.74±0.08(214)c	16.03±0.08(225)b	14.92±0.14(180)a	15.07±0.18(57)a	
Pupa (day)	19.86±0.09(95)f	14.53±0.06(95)e	9.48±0.04(216)d	8.82±0.03(207)c	6.64±0.03(223)b	6.19±0.04(167)a	6.00±0.00(12)a	
Adult longevity (day)	9.88±0.33(95)d	10.60±0.31(95)d	10.26±0.15(216)d	8.79±0.15(207)c	7.79±0.14(223)b	6.50±0.11(167)a	6.25±0.28(12)a	
Egg-pupa (day)	106.33±1.22(95)g	70.11±0.82(95)f	39.13±0.15(216)e	34.56±0.09(207)d	25.67±0.09(223)c	24.06±0.15(167)b	21.92±0.48(12)a	
Total generation (day)	116.21±1.29(95)g	80.71±0.90(95)f	49.40±0.21(216)e	43.35±0.20(207)d	33.47±0.17(223)c	30.56±0.19(167)b	28.17±0.68(12)a	
5th instar mass (mg)	6.86±0.32(163)a	13.71±0.99(107)b	31.94±0.68(235)c	45.40±0.66(223)e	49.72±0.90(228)f	41.53±1.14(183)d	14.20±0.70(212)b	
Pupa mass (mg)	74.86±1.54(121)b	94.08±2.07(97)e	83.61±1.39(226)cd	87.19±1.40(209)d	82.42±1.29(225)c	80.02±1.56(177)bc	48.09±1.90(56)a	

Note: Data in the table are given as the mean ± SE. The numbers in () represent the sample size, and different lowercase letters on the same row indicate significant differences between temperatures ($p < 0.05$).
